# Organizational and Institutional Response to the COVID-19 Pandemic

**DOI:** 10.1177/00027642231155373

**Published:** 2023-02-14

**Authors:** Jeremy Schulz, Laura Robinson, Maria Laura Ruiu, Apryl Williams

**Affiliations:** 1 Institute for the Study of Societal Issues, UC Berkeley, Berkeley, CA, USA; 2 Department of Sociology, Santa Clara University, Santa Clara, CA, USA; 3 Department of Criminology and Sociology, Northumbria University, Newcastle-upon-Tyne, Tyne and Wear, UK; 4 Department of Communication and Media, University of Michigan, Ann Arbor, MI, USA

**Keywords:** organizations, institutions, COVID-19, carceral institutions, crisis response

## Abstract

This issue of *American Behavioral Scientist* deals with the various ways in which different kinds of organizations cope with the manifold challenges brought about by the COVID-19 pandemic. Together, these articles map the challenges and opportunities encountered by a variety of organizations in a major public health crisis. The first section of the issue takes up the theme of adaptive crisis response in relation to two different kinds of organizations. This section begins with a comprehensive overview of U.S. nonprofit organizations’ responses to the COVID-19 pandemic. The second article expands on the theme of communication practices in organizations using digital communication platforms which facilitate constructive forms of disagreement or “creative conflict.” Both of these articles indicate the potential positive outcomes of entrepreneurial organizational response. In the next section, we turn to organizational responses hampered by digital inequalities. The first article addresses digital inequalities and eLearning during the pandemic in the country of Pakistan. The next article also uses a digital inequalities framework to probe infrastructural inadequacies faced by the criminal justice system in terms of hindrances to external communication for incarcerated populations during the pandemic. This pair of articles underscores the importance of infrastructure as a necessary element of successful crisis response. The third section of the issue continues with case studies of carceral institutions with the first article offering insight into strategies used by incarcerated people to generate a sense of normality despite pandemic disruptions. Finally, the issue closes with an article revealing the delicate balancing act which rural U.S. law enforcement carried out when competing imperatives made it extremely difficult to manage public health and public safety simultaneously.

## Organizations and Communication During the Pandemic

This issue of *American Behavioral Scientist* deals with three themes illuminating the diverse ways in which different kinds of organizations attempted to cope with the manifold challenges brought about by the COVID-19 pandemic. Together, these articles map the challenges and opportunities encountered by a variety of organizations in a major public health crisis.

## Adaptive Crisis Response

The first section of the issue takes up the theme of adaptive crisis response in relation to two different kinds of organizations. The special issue’s initial contribution seeks to explain how nonprofit organizations reacted to the challenges and opportunities presented by the pandemic in terms of the degree to which these organizations followed one of three competing strategies: innovation, retrenchment, or perseverance. This article, entitled “U.S. Nonprofit Organizations Respond to the COVID-19 Crisis,” examines these three crisis response strategies as a function of organization-level determinants, including organizations’ communication practices and levels of resources. To do so, the authors enlist a unique dataset pertaining to U.S.-based nonprofit organizations. Analyzing the responses of these nonprofit organizations relative to three foundational strategies, innovation, perseverance, and retrenchment, the quantitative analysis shows that higher levels of innovation stem from higher levels of resources and communication with external constituencies. Retrenching organizations, on the other hand, stood out on account of their relative lack of communication with external constituencies. Among its many contributions, this article points out new directions for the study of organizational crisis response among nonprofit organizations, with special attention to the strength of the communicational ties between organizations and their environments.

Continuing the theme of innovative organizational response, the next article also deals with the intersection of communication practices and institutional domains, except it takes a more micro-perspective on this theme. In this article, entitled “COVID-19, Creative Conflict, and the Seven Cs: A Social Diagnosis of Digital Communication Platforms for Gen Z/Gen T,” the analytical focus is the ways in which particular digital discussion platforms facilitate constructive and collegial discourse, even in the face of substantive disagreements. Using rich qualitative data, this contribution highlights the potential for text-based discussion fora to promote constructive discourse provided that they meet particular design criteria. Contrary to much dismissive coverage of online communication platforms, the evidence in this study shows that for members of Gen Z/Gen T, well-designed text-based communication platforms can foster thoughtful and civil exchanges on potentially controversial topics. Such fora encourage more meaningful exchanges which allow a variety of benefits including a diversity of views, reduction of acrimony, and a willingness to listen to dissenting opinions. The implications of such constructive exchanges for the workplace are explored, as this environment is one where prosocial behavior can spell the difference between successful collaborations and organizational success, on the one hand, and destructive conflict and organizational failure, on the other hand.

## Digital Inequalities and Organizational Response

From organizations employing digital resources to respond to the pandemic, in the next section, we turn to organizational response that was hampered by digital inequalities. The next article addresses the impact of digital inequalities on educational organizations in the developing world. Entitled “The COVID-19 Pandemic and E-learning: The Digital Divide and Educational Crises in Pakistan's Universities,” this research provides an overview of the digital access and resource inequalities across different universities in Pakistan struggling to continue to serve their students in the early stages of the pandemic. The article maps the profound consequences of inadequacies of the digital infrastructure, inadequacies which were exacerbated by the pandemic. Pointing to the gap between policy mandates and infrastructural realities, the study finds that neither the vast majority of students nor teachers in Pakistan had sufficient digital resources to implement the eLearning initiatives mandated by the government. In examining eLearning and digital inequalities in an understudied case, the article offers important insights into organizational response in developing economies but also reminding us of variation within and across populations. As the authors make clear, even among important organizational organizations, the pandemic exposed deep rifts between the digital resources enjoyed by affluent urbanites and less advantaged populations in the same country.

The next article probes also uses a digital inequalities framework and continues the call to enhance access to digital communication tools for incarcerated populations in “Locked in and locked out: How COVID-19 is making the case for digital inclusion of incarcerated populations.” This article makes the case that the lack of adequate connectivity within this already profoundly disadvantaged group was thrown into particularly sharp relief amidst the pandemic. During this crisis, face-to-face contacts between incarcerated individuals and members of the public were sharply curtailed in the interest of public health, but without any mitigating measures such as the expansion of digital connectivity. Indeed, in comparing different carceral regulations, the articles make the importance of better regulated and equalized internet access within this population apparent. As the study contends, it is critical that U.S. carceral facilities supply digital communications both as compensatory channels in the face of public health emergencies and also because internet experience and skills play important roles in smoothing reentry into mainstream society and reducing recidivism.

## The Criminal Justice Systems and Institutions of Social Control

The third section of the issue continues to examine organizational response among total institutions. In the next contribution dealing with institutions of social control, the focus is on the eating practices of incarcerated individuals in the New York system during the pandemic lockdown orders. This article is entitled “Prison, Material-Organizational Bricolage, and Precarious Frameworks of Normality in an Era of Disruption.” It deploys a rich trove of documentary evidence and interviews to demonstrate how incarcerated individuals improvised in often ingenious ways in order to procure the necessities to create a meal for themselves during the particularly strict COVID-19 lockdowns. Many of the accounts referenced in the article come from the Thanksgiving dinner which incarcerated individuals prepared and served with the aid of many mundane objects—such as buckets—which required special effort to secure because “care packages” from the outside world were restricted. The resourcefulness with which they improvised—within an environment of a particularly restrictive lockdown—indicates the resilience with which these individuals met particularly difficult effects of the pandemic with resourceful improvisation during a time of crisis.

Continuing the focus on organizations and institutions in the arena of criminal justice, the issue closes with “The Fine Line: Rural Justice, Public Health and Safety, and the Coronavirus Pandemic.” This article explores the issues of public health and safety as they unfolded in a rural region in the Pacific Northwest. The analysis is grounded in a combination of news articles and press releases, sheriff’s department Facebook posts, publicly available jail data, courtroom observations, in-depth interviews with those who have been held in rural jails, and interviews with rural law enforcement staff. As the study shows, the pandemic subjected local law enforcement and the justice system as a whole to conflicting cross-pressures. On the one hand, rural law enforcement officials are often forced to implement public health measures that may run counter to the political ideologies of those who elected them to serve, namely politically conservative local populations concerned with perceived threats to individual “freedoms.” Simultaneously, local law enforcement are expected to employ punitive measures to control perceived criminal activity. Given the community health restrictions occasioned by the pandemic, however, these officials found themselves tasked with enforcing health and safety regulations with respect to both incarcerated population and the public at large. This goal conflicted with the tradition of maintaining public health through informal social norms. The study finds that the health mission of local law enforcement was often subordinated to the imperative of appearing tough on crime and supportive of personal liberty, when the two imperatives came into conflict.

